# The effect of sources and air mass transport on the variability of trace element deposition in central Poland: a cluster-based approach

**DOI:** 10.1007/s11356-017-9932-2

**Published:** 2017-08-19

**Authors:** Patrycja Siudek, Marcin Frankowski

**Affiliations:** 1 National Marine Fisheries Research Institute, Kołłątaja 1 Street, 81-332 Gdynia, Poland; 2 Department of Water and Soil Analysis, Faculty of Chemistry, Adam Mickiewicz University in Poznań, Umultowska 89b Street, 61-614 Poznań, Poland

**Keywords:** Urban area, Anthropogenic sources, Trajectory, Trace elements, Bulk deposition, Rainwater, Cluster analysis

## Abstract

Measurements of trace element (As, Cu, Cd, Cr, Ni, Pb, Zn) deposition fluxes were conducted simultaneously in two contrasted environments, i.e., urban and forest, between April 2013 and October 2014. This was the first such project in central Poland, aimed at long-term observations of trace elements in the atmosphere and their distribution, transport, and deposition pattern. The receptor sites were different in terms of local meteorological conditions, emission potential, and distance to major anthropogenic sources. The deposition fluxes of all trace elements showed clear seasonal variations, with relatively higher values in winter than in summer. The main factors affecting interannual differences in concentrations and deposition of trace elements in central Poland were local emission from industrial and commercial sources, and changes in atmospheric conditions (wind speed and direction, boundary layer, precipitation amount, air mass origin). In this study, the impact of regional and long-range transport on trace element deposition was determined using the air back-trajectory cluster analysis. During the summertime of 2013 and 2014, the predominant SW and E advections from regional and remote anthropogenic sources in Europe were responsible for high deposition of Cd, Cr, Pb, Cu, and Zn, whereas during the wintertime of 2013/2014, we observed a significant influence of polluted air masses from southeastern regions. Based on the Pb/Zn ratio, it was found that regional sources significantly influenced the aerosol composition and rainwater chemistry within the study domain. However, the role of a long-range transport of anthropogenic pollutants was also important. In addition, a relatively small difference in the Pb/Zn ratio between both sites (urban 0.26 ± 0.18, forest 0.23 ± 0.17) may suggest (1) very similar contribution of anthropogenic sources and (2) minor importance of atmospheric transformation processes of these metals in the aqueous phase.

## Introduction

The atmospheric budget of trace elements (TEs) is controlled by two major processes: emissions from various anthropogenic/natural sources and deposition via wet and/or dry scavenging, including the in-cloud and below-cloud mechanisms. The removal of trace elements from the atmosphere seems to be of crucial importance for the aquatic and terrestrial environments due to toxicity, bioaccumulation, and carcinogenic properties of these metals. It has been previously demonstrated that heavy metals may cause serious environmental problems, i.e., soil damage, contamination of water, negative biological effects on plants, biota, air quality, and public health (Fernández-Espinosa and Rossini Oliva 2006). Many studies have highlighted that the atmospheric deposition of metallic compounds depends on numerous meteorological factors and atmospheric processes; however, special emphasis is given on different anthropogenic sources of these pollutants (e.g., fossil fuel combustion, residential heating, non-ferrous metal production, traffic emissions, road dust re-suspension, biomass burning, non-exhaust traffic emissions; Conko et al. 2004; Connan et al. 2013; Lynam et al. 2013; Moreda-Piñeiro et al. 2014; Guo et al. 2015; Dong et al. 2015, 2017). In this aspect, the receptor-based source apportionment models have been extensively applied in order to estimate the predominant influence of these sources on TE concentrations in different sites. There is a broad range of dependencies between trace metal emissions and formation processes, as well as between atmospheric conditions and the removal rates via dry deposition/precipitation. A number of studies have shown the spatiotemporal variability of TEs in wet (Sakata and Asakura 2009; Montoya-Mayor et al. 2013; Kara et al. 2014; Tripathee et al. 2014; Guo et al. 2015; Lynam et al. 2015; Pan and Wang 2015) and dry (Gunawardena et al. 2012; Connan et al. 2013; Lynam et al. 2013) deposition.

In recent years, the receptor methods based on backward-trajectory cluster analysis have been extensively applied to quantitatively determine the impact of source regions on the urban background monitoring sites in Amsterdam, Athens, Birmingham, and Helsinki (Kavouras et al. 2013). Moreda-Piñeiro et al. (2014) used this approach to classify air mass trajectories into five groups, representing the major areas with a significant contribution of trace metals to rainwater at the suburban site in the northwestern coast of Spain. Although there is a growing concern about urban air quality, environment, and human health, the integrated and long-term regional studies dedicated to trace elements are still limited in some countries of Central Europe.

Due to the lack of data on the bulk deposition of trace metals in central Poland, this study examines the influence of emission sources and regional-scale transport on TEs in precipitation. The measurements were carried out simultaneously in two contrasted environments (urban and forest), between April 2013 and October 2014. The aim of the present study was to evaluate the seasonal variability of TE concentrations and their deposition fluxes. We examined the influence of local, regional, and long-range transport on seven trace elements (arsenic (As), Cu, Cd, Cr, Ni, Pb, and Zn) in rainwater samples and investigated the contribution of different source regions. Additionally, the cluster-based approach was used to elucidate the differences between nine transport vectors. Finally, we compared our results with other worldwide observations.

## Materials and methods

### Experimental domain

**Fig. 1:**
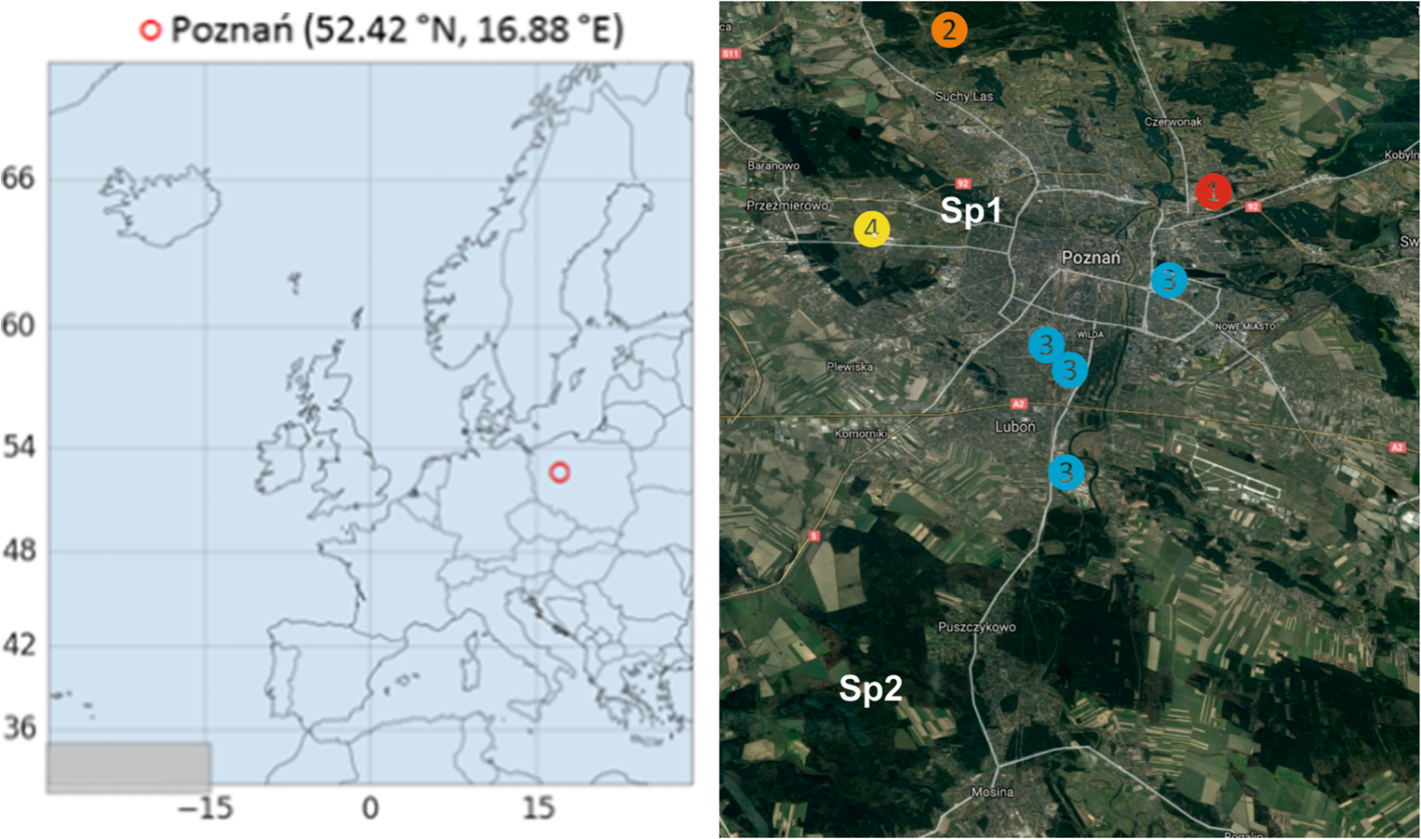
Map of the study domain and surrounding area in the Wielkopolska Province (red circle), central Poland. The right image is enlarged to show two sampling areas, Sp1 (urban) and Sp2 (forest). The color-numbered dots show major local sources (1 red, coal-fired Karolin power plant; 2 orange, municipal solid waste management and recycling; 3 blue, industrial units producing metals and paints, and chemical plants; 4 yellow, international airport)

The concentrations of trace metals in rainwater and their bulk deposition fluxes were investigated at two different sampling sites (urban and forest) in the Wielkopolska Province of central Poland (Fig. 1).

The urban sampling site (Sp1; 52.42° N, 16.88° E) was located approximately 3 km northwest of the Poznań City Center. The surroundings of this place are dominated by a botanic garden, commercial/residential buildings, and roads. In addition, about 4 km east of this site, there is an international airport (Poznań-Ławica), and about 10 km southwest of the first sampling location, there is a large coal-fired Karolin power plant. There are also other sources within a 20-km radius such as industrial units producing metals and paints, waste incinerators, municipal solid waste incineration, construction sites, and domestic sewage. The urban measurement site was located in a traffic-impacted area, ca. 300 m W-SW from two streets (Dąbrowskiego Street and Saint Wawrzyńca Street; mean density of traffic 15 × 10^3^ vehicles per day).

The forest sampling site (Sp2; 52.26° N, 16.80° E) was located at the Ecological Station of Adam Mickiewicz University in Jeziory, which is a small village situated 30 km southwest of the Poznań City center. This Ecological Station is situated on the morainic plateau covered by mixed pine-oak forest. There are no urban/industrial activities that produce large loadings of local pollutants in the vicinity of this station. Moreover, the traffic emission is relatively low (medium-traffic road is ca. 4 km away).

### Sampling method

At both sampling sites, the sampling set consisted of an acid-washed polyethylene funnel (*Ø* = 36 cm, reception surface = 0.0962 m^2^) connected with an acid-cleaned borosilicate bottle by a Teflon adapter. The bulk sampler was deployed 1.5 m above ground level in an open area of each site. According to the standards, the sampling system was installed far from buildings and trees to ensure optimal measurement conditions. Additionally, to prevent the collected samples from solar radiation, each sampling set was equipped with an outer wooden tube. The sampling time was different for each experiment; however, in most cases, the experiments were finished immediately or no longer than 6 h after precipitation, to reduce the possible artifacts caused by leaves, insects, and adsorption of rainwater compounds on the sampler surface. At both sites, the sampling sets were manually changed after a precipitation event. Each collected sample was directly transported to the analytical laboratory located at the Adam Mickiewicz University in Poznań, and then a new acid-cleaned bottle was loaded to the funnel surface that had been previously rinsed with double-deionized water.

### Sample analysis and quality control

The rainwater samples were collected over a 1.5-year study period, between April 2013 and October 2014. Each sample was attributed to one of the sampling seasons: spring 2013 (April–May), summer 2013 (June–August), fall 2013 (September–November), winter 2013/2014 (December–February), spring 2014 (April–May), summer 2014 (June–August), and fall 2014 (September–October). The method detection limits (MDLs) for rainwater samples calculated as three times the standard deviation of the replicate measurements of a blank solution were as follows: graphite furnace atomic absorption spectrometry (GF-AAS): Cd, 0.003 μg L^−1^; Cr, 0.02 μg L^−1^; Cu, 0.03 μg L^−1^; Ni, 0.1 μg L^−1^; Pb, 0.03 μg L^−1^; and As, 0.1 μg L^−1^, and flame atomic absorption spectrometry (F-AAS): Zn, 2.0 μg L^−1^. The concentration levels of Cu, Cd, Ni, Cr, As, and Zn were, in some cases, below the MDLs. The relative standard deviation (RDS) for triplicate analysis of trace metals did not exceed 5 and 7% for GF-AAS and F-AAS, respectively.

The analysis of the following trace elements: Cr, Pb, Mn, Ni, Cd, and As in rainwater samples was performed using an atomic absorption spectrometer (AA-7000 Shimadzu, Japan) with graphite furnace atomization, according to the US EPA method no. 200.7:2001. The rainwater Zn concentration was determined quantitatively through the flame atomization spectrometry (AA-7000 Shimadzu, Japan). Additionally, the palladium matrix modifier Pd(NO_3_)_2_ was used to maintain high analytical sensitivity during the Cd, Pb, and As measurements with GF-AAS. Details regarding the analytical procedures and instrument setup can be found elsewhere (Siudek et al. 2015). The quality assurance procedures were routinely controlled using standards, duplicate blanks, and sample blanks (bottles filled with double-deionized water to correct the results for possible contamination). The measurement precision represented less than 5% of the measured sample concentration.

During the 19-month study period, the total precipitation amount in Poznań was 943.3 mm. The daily precipitation amounts in millimeters were measured using a Hellman rain gauge. At the urban site, the average monthly precipitation ranged between 5.6 mm (February 2013) and 109.8 mm (June 2013), with an average of 46.6 ± 27.2 mm per month. The physicochemical parameters, i.e., pH and electric conductivity (EC) of an unfiltered rainwater sample, were determined by a Mettler Toledo pH meter. At the urban site, the pH of rainwater samples varied from 3.61 (fall 2013) to 7.46 (spring 2013), with a mean ± SD of 5.65 ± 0.72. At the forest site, the pH values ranged between 3.81 and 7.00. The range of EC for rainwater samples collected at the urban site was 5.38–151.6 μS cm^−1^, and at the forest site, 5.61–109.2 μS cm^−1^.

### Meteorological data analysis

The Statistica 10.0 software was used to perform descriptive statistics and regression analysis for all data. The trace element concentrations and deposition fluxes were tested for normality (Shapiro-Wilk test), outliers (Grubbs’ test), and distribution pattern (Levene’s test). The statistically significant differences in mean concentrations were established using parametric (ANOVA) or non-parametric (Kruskal-Wallis) tests. Spearman’s regression analysis at *p* value below 0.05 was used to find linear correlations between trace metals.

The meteorological parameters, i.e., air temperature, atmospheric pressure, relative humidity, wind speed, and direction, were registered by the automatic local weather station located at the Ecological Station of Adam Mickiewicz University in Jeziory and at the Botanic Garden in Poznań (measurement data not available via a Web site). Additionally, to estimate the regional, macro-regional, and distant source areas of TEs, we used data retrieved from the HYSPLIT model (Draxler and Rolph 2003). This model allowed to compute air parcel trajectories with the information about their origin. In the present study, we plotted 2-day air mass backward trajectories from the starting heights of 500, 1000, and 1500 m above ground level using the archive meteorological GDAS database provided by NOAA (Draxler and Rolph 2003). Such approach allowed to track transport processes that affect trace element deposition in the atmosphere over the study domain. The trajectory simulations were generated for every day, for the following hours: 00:00, 06:00, 12:00, and 18:00 UTC, between April 2013 and October 2014. Results from these simulations were used in the trajectory cluster analysis to examine the air mass origin and long-range transport of TEs over the study domain. Following these calculations, the hierarchical cluster analysis of backward trajectories was performed to extract only those clusters which were attributed to a prevalent transport pattern during the sampling period. Specifically, the total spatial variance (TSV) parameter was used to establish the optimum number of clusters. This parameter is referred to the sum of the squared distances between endpoints along trajectories and the mean of the trajectories within each cluster. Here, by clustering the backward trajectories, it was possible to distinguish various groups (S, SW, SE, N, NE, NW, and L as local) which represented major source areas of markedly different emission potential. The characteristics of the selected clusters are discussed in the next section.

## Results and discussion

### Trace element concentrations in rainwater samples

As shown in Table 1, the highest mean concentrations in rainwater samples from the urban site were determined for Zn followed by Cu > Pb > Ni > As > Cr and Cd, whereas in the samples from the forest site, trace metal concentrations decreased in the following order: Zn > Pb ≥ Ni > Cu > As > Cr > Cd. The rainwater Zn concentrations ranged from < MDL to 137.1 μg L^−1^, with a mean of 19.4 μg L^−1^ at the urban site, and from < MDL to 100.5 μg L^−1^ at the forest site (mean 14.3 μg L^−1^, Table 1). The mean Zn values were comparable to the results obtained by Guo et al. (2015) and Sakata and Asakura (2009) (14.2 and 12.0 μg L^−1^, respectively). However, slightly lower concentrations of Zn were observed in rainwater samples collected at a rural site in Europe (Connan et al. 2013), and some other regions of Canada (Lynam et al. 2015) and the USA (Conko et al. 2004).

**Table 1:** Comparison of mean concentrations and ranges (in parentheses) of trace metals in rainwater from this study and from other regions (see references in the table).

Sites	References	As	Cd	Cr	Ni	Pb	Cu	Zn
Poznań, Poland	This study	*1.13* (< MDL–7.14)	*0.06* (< MDL–0.40)	*0.53* (0.06–2.92)	*3.65* (< MDL–24.08)	*4.16* (0.32–30.8)	*8.22* (0.29–49.1)	*19.4* (< MDL–137.1)
Jeziory, Poland	This study	*1.50* (< MDL–7.73)	*0.06* (< MDL–0.55)	*0.42* (0.10–2.79)	*3.43* (< MDL–17.57)	*3.54* (0.32–18.7)	*2.83* (0.12–10.1)	*14.3* (< MDL–100.5)
Lhasa, Tibet	Guo et al. (2015)	*0.64*	*0.028*	*0.43*	*0.58*	*1.59*	*1.71*	*14.2*
Reston, Virginia, USA	Conko et al. (2004)	*0.1* (0.05–1.0)	*0.06* (0.01–0.45)	*0.25* (0.10–0.50)	*0.35* (0.10–1.5)	*0.54* (0.26–3.1)	*0.95* (0.20–5.4)	*5.5* (2.0–15)
A Coruña, Spain	Moreda-Piñeiro et al. (2014)	< MDL^a^	–	*0.28* (0.08–1.9)^a^	*1.0* (0.23–3.8)^a^	*0.51* (0.08–1.5)^a^	*2.1* (0.44–10.5)^a^	*55.7* (15.5–145)^a^
Marais Vernier, France	Connan et al. (2013)	–	(< 0.01–0.06)	–	(0.3–1.15)	(0.08–0.73)	–	(2.4–22.9)
Patricia McInnes, Canada	Lynam et al. (2015)	*0.15*	–	–	–	*0.3*	*1.2*	*5.8*
Nakanato, Japan	Sakata and Asakura (2009)	*0.71*	*0.14*	*0.18*	*0.65*	*4.6*	*0.81*	*12*
Nanjing, China	Tang (2007)	*5.0*	*3.3*	*10.6*	*1.4*	*13.1*	–	*28.2*
Singapore, Singapore	Hu and Balasubramanian (2003)	–	*0.33*	*1.62*	*3.86*	*7.23*	*5.58*	–
Laohugou, Northern Tibetan Plateau, China	Dong et al. (2015)	–	*0.024* ^b^	*0.654* ^b^	*1.232* ^b^	*0.099* ^b^	*0.479* ^b^	*0.712* ^b^
Western Qilian Mountains, China	Dong et al. (2017)	–	*0.008*	*0.941*	*0.206*	*0.077*	*0.302*	*0.061*
Mt. Nyainqentanglha region, China	Huang et al. (2013)	–	*0.022* ^b^	*1.989* ^b^	*2.743* ^b^	*2.140* ^b^	*5.275* ^b^	*18.703* ^b^

All data are given in micrograms per liter

*MDL* method detection limit

^a^Data given for soluble fraction

^b^Data given for surface snow and snowpit samples

The mean values are presented in italic font﻿

Furthermore, the concentrations of Zn and other trace elements measured in this study revealed relatively higher levels than those registered in rainwater samples collected in western Qilian Mountain (Dong et al. 2017) and those in surface snow samples from remote alpine glaciers in the northern Tibetan Plateau (Dong et al. 2015). As can be seen in Table 1, the concentrations of Zn and Ni in rainwater samples collected at the urban site in Poland were very similar to the results obtained for snowpit samples from Mt. Nyainqêntanglha, southern Tibetan Plateau (Huang et al. 2013). In contrast, much higher values of Zn in precipitation, as compared with data from the present study, were registered at the urban sites in China (Hu and Balasubramanian 2003; Tang 2007). The relatively high Zn concentrations measured in rainwater from Poznań might be attributed to different local/regional sources and long-range transport from the adjacent polluted regions. There were several potential local sources of Zn in the atmosphere of the urban study domain (Fig. 1). They were related to industrial activities (e.g., public electricity and heat production, production of chemicals, waste incineration), road/rail transportation (tire and brake wear, re-suspension of road dust), residential sector (domestic heating), agricultural waste burning, and direct emission from polluted soils. Recent biomonitoring studies by Fantozzia et al. (2013) have revealed high concentrations of Pb, Zn, Cu, and Cd in different compartments such as topsoil, plant leaves, and tree canopy of the urban park of Siena (Italy). Also, measurements by Kowalski and Frankowski (2016) have highlighted elevated concentrations of Hg species in different vegetation types from the Poznań agglomeration, caused by multiple traffic-related pollutants and re-suspension processes. Therefore, high concentrations of zinc measured in rainwater samples from this study were likely related to the anthropogenic emission from different local/regional sources.

Cu is known as a trace element associated with industrial pollution, vehicle emission, primary non-exhaust traffic emission, and road dust (Tian et al. 2016). In this study, the maximum Cu concentration in urban rainwater samples was almost four times higher than the values measured at the forest site (Table 1). The observed differences in rainwater Cu concentrations between urban and forest areas can be explained by different emission potential of these contrasted environments, especially contribution from sources related to vehicular traffic and industrial coal combustion.

The concentrations of Pb and Ni measured in this study were relatively higher than those observed by Guo et al. (2015). Also, previous measurements in heavily polluted industrial regions of South Asia revealed high Pb, Cr, and Cd concentrations in rainwater samples (Hu and Balasubramanian 2003; Tang 2007). As shown in Table 1, the Cr concentration range in rainwater samples from this study was comparable for both sites (0.06–2.92 μg L^−1^ for Poznań and 0.10–2.79 μg L^−1^ for Jeziory). Also, the concentrations of Cd did not reveal a wide range, and the mean value did not exceed 0.06 μg L^−1^ at both sites. In this study, rainwater samples were also moderately enriched in As as compared with data from several other sites (Guo et al. 2015; Conko et al. 2004; Moreda-Piñeiro et al. 2014; Lynam et al. 2015; Sakata and Asakura 2009). This carcinogenic metal exhibited a relatively wide range of concentrations at both locations, with quite different mean values during the parallel campaigns, i.e., 1.50 μg L^−1^ for forest and 1.13 μg L^−1^ for urban (Table 1).

The observed spatiotemporal variability in trace element concentrations can be partly explained by different rainwater solubility potentials of these metals. Moreda-Piñeiro et al. (2014) found that trace elements in rainwater samples revealed a complex dilution effect, ranging from 10.5 to 98.1%. They also showed that pH of rainwater did not significantly affect the solubility of Al, Ba, Co, Cu, Fe, Mn, Ni, Sr, and V, in contrast to Cr and Pb. Conko et al. (2004) have stated that the quantity and solubility of trace elements in rainwater should be considered for an individual precipitation event. They also pointed out that rainwater samples from suburban areas might contain a higher amount of trace elements in particulate phase compared with urban sites. Furthermore, Varga et al. (2007) demonstrated that the compounds characterized by low solubility have a minor effect on water activity in the early stage of droplet formation. It this study, we did not estimate the phase partitioning of trace elements between soluble and particulate fractions; however, we suppose that the solubility effect of TEs might give fairly similar results to those presented in the study by Conko et al. (2004). This issue will be undertaken in the follow-up studies.

### Pb/Zn ratio as a marker of short-range (local, regional) and long-range transports

**Fig. 2:**
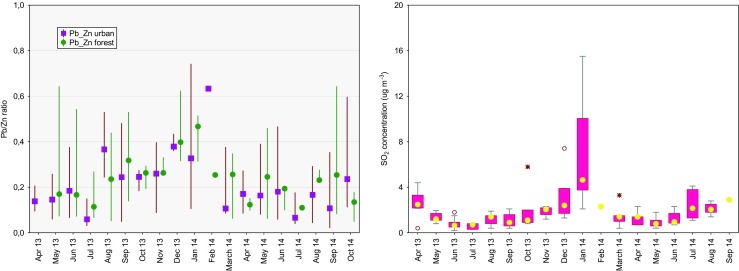
The Pb/Zn concentration ratio as a function of season and site; urban: *F*(18;105) = 2.45, *p* = 0.00; KW-H(18;124) = 39.36, *p* = 0.00; forest: *F*(17;59) = 0.57, *p* = 0.90; KW-H(18;78) = 0, *p* = 0.00. The outliers are not shown. The right image displays the airborne SO_2_ concentration at the urban site in Poznań (data from the Voivodship Environmental Protection Fund in Poznań)

It has been shown that combustion processes are key sources of an airborne fraction of trace elements such as Pb, Zn, Cu, As, and Cd (Hławiczka et al. 2001; Murphy et al. 2007; Juda-Rezler and Kowalczyk 2013). The Pb/Zn ratio has been suggested by Sakata and Asakura (2009) and other authors (e.g., Okuda et al. 2004) as a suitable tracer of industrial pollution, especially for the sites impacted by long-range transport from highly polluted regions. In addition, some previous works showed that trace element ratios obtained by a linear regression approach can be applied to the assessment of short-range transport and source contribution in the urban atmosphere (Murphy et al. 2007; Lin et al. 2015). More recent studies by Rossi et al. (2017) have examined the changes in sediment molar Pb/Zn ratios to infer the influences of particulates from different metallurgical facilities (i.e., smelter, zinc works, steel, and wire works). According to the abovementioned studies, we examined the ratio of these two combustion products in rainwater samples collected in this study. Figure 2 displays the monthly fluctuation of Pb/Zn ratio in Poznań and Jeziory. At the urban site, the monthly cycle of airborne SO_2_ concentration generally showed a similar trend compared with the Pb/Zn ratio, indicating a large contribution from combustion processes during the wintertime study period (Fig. 2, right). The mean concentration ratio of rainwater Pb/Zn in the forest area was 13% higher than that at the urban site. At the forest site, the concentration ratio of Pb to Zn in rainwater samples ranged between 0.01 and 0.93, whereas at the urban site, the Pb/Zn ratio ranged between 0.05 and 0.89. As can be seen in Fig. 2, the Pb/Zn ratio exhibited a clear seasonal variability for both sites during the study period, with higher values during the winter of 2013/2014 and much lower during the summer and spring of 2014. Surprisingly, the highest value of the Pb/Zn ratio for the urban and forest sites was observed in different months, i.e., in January 2014 and May 2013, respectively.

At the urban site, the peak Pb/Zn ratio was observed during the coldest month of the study period and was directly influenced by an increase in coal combustion for household heating. In Poland, the hard coal is largely used in commercial coal-fired, residential and industrial sectors and contains different amounts of Pb (14.30–25.00 ppm) and Zn (38.40–69.00 ppm) (Juda-Rezler and Kowalczyk 2013). Therefore, a significant amount of Pb and Zn was probably emitted to the atmosphere during the combustion of coal and then incorporated in the airborne fraction. The study by Kryza et al. (2010) provided evidence that a large amount of SO*x* originating from a household sector in Poland can be atmospherically deposited in polluted areas of large cities (e.g., Poznań) where residential, commercial, and agricultural sources dominate. This finding is consistent with the data presented in Fig. 2.

Other possible reasons for the observed high value of the Pb/Zn ratio at this site in January and February 2014 were atmospheric conditions that favored the accumulation of emitted pollutants over the study domain (e.g., boundary layer dynamics, lower mixing layer height, thermal inversion, local transport processes). The wind speed analysis revealed the occurrence of frequent low and medium winds from the S-SW in January 2014. Thus, local anthropogenic emissions from the nearby two three-story buildings equipped with wood- and coal-based heating systems and small space heaters with no emission controls might have caused high loadings of combustion products in the atmosphere. At the forest site, the highest Pb/Zn in May 2014 was strongly influenced by transport processes, including effects of urban plumes from Poznań agglomeration and regional advection of polluted air masses from industrially impacted regions, i.e., southern Poland, Western Europe.

In the present study, zinc was the dominant trace metal in bulk deposition at both sites; however, higher Zn levels were more frequently measured in urban rainwater samples than in those collected at the forest site. In Poznań, we observed high positive correlations (*R* ^2^ > 0.700) for Pb-Cd, Pb-Cu, and Zn-Cd and slightly lower linear correlations for Pb-Cr (0.649), Pb-Zn (0.640), Cu-Zn (0.640), Cu-Cr (0.539), Zn-Cr (0.517), and Cu-Cd (0.505), suggesting a significant impact of coal-fired power plants and other industrial processes on TE interannual variation (Table 2). In Poland, large emission of As and Pb is attributed to the combustion processes in industrial sector, whereas the elements such as Cd, Cr, and Ni are largely emitted from coal combustion in the residential sector (Juda-Rezler and Kowalczyk 2013). The mentioned authors showed that trace element concentrations in bituminous coals from Polish coalmines are on average 52, 0.1, 27, 18, 19, and 57 ppm of As, Cd, Cr, Ni, Pb, and Zn, respectively. At the second site, the significant correlations between trace elements in rainwater samples were also found; however, the *R* ^2^ greater than 0.700 was noted only for Pb and Cd, indicating a common anthropogenic origin of these metallic compounds (Table 2).

**Table 2:** Correlation matrix for trace elements in rainwater collected at the urban (data in italics and bold-italics) and forest sites in central Poland, between April 2013 and October 2014

	As	Cd	Ni	Cr	Pb	Cu	Zn
As		*0.419*	*0.033*	*0.057*	*0.292*	*0.152*	*0.236*
Cd	0.419		*0.164*	*0.346*	***0.706***	***0.505***	***0.746***
Ni	0.260	0.416		*0.063*	*0.136*	*0.166*	*0.146*
Cr	0.218	0.480	0.439		***0.649***	***0.539***	***0.571***
Pb	**0.597**	**0.705**	0.481	**0.547**		***0.708***	***0.640***
Cu	0.301	0.401	0.446	0.423	**0.502**		***0.640***
Zn	**0.597**	0.287	0.043	0.058	0.369	0.174	

The bold and bold-italic data represent high and moderate correlations (0.500–0.700)

In Poland, the energy consumption of hard coal in 2014 was 72.3 × 10^6^ tons (Central Statistical Office 2016). About 60% of this amount was used in the energy and heat industry sector (e.g., power plants, heat and power plants, non-professional and professional heating plants), 25% in industrial and construction companies, and 13% by retail customers (households, agricultural holdings, etc.). It should be noted that the structure of household energy consumption per one habitant in Poland significantly differs from those observed in the EU. Specifically, the hard coal is treated as a main source of energy in Poland, its consumption accounts for about 32%, whereas in other EU countries, it is on average 3% (Central Statistical Office 2016).

As mentioned before, the winter study period was characterized by high Pb/Zn ratios. This suggests that the urban atmosphere was largely affected by local/regional point emission sources mostly related to high-temperature combustion processes. The large contribution of these sources was confirmed in past studies (Siudek et al. 2015, 2016). Briefly, during the heating season (October–March), heavy metals within the urban study area originated mostly from distinct anthropogenic sources such as industrial activities (coal-fired Karolin power plant), non-fully controlled combustion in domestic heating units, residential wood burning, and local transport (vehicle exhaust emission, brake wear, tire abrasion, road dust re-suspension). This hypothesis can be also supported by previous measurements of trace element content and size distribution of particulate matter emitted from coal combustion in residential furnaces in Poland (Hławiczka et al. 2001). It was found that almost 80% of particulate matter was emitted as a < 12-μm-size mode, whereas the partition factors (defined as distribution of metal streams between the feed coal and its combustion products emitted to the atmosphere) for Zn, Pb, and Cu were 0.59, 0.33, and 0.34, respectively. Also, high deposition fluxes of Zn during winter in Poznań could be associated with a specific meteorological situation (e.g., thermal inversion, lower mixing height, low atmospheric turbulence, weaker photochemistry) that mitigated the diffusion of pollutants, and resulted in more complex atmospheric chemistry of urban plumes. Such kind of significant relationships between local anthropogenic emissions and meteorological variables have been previously observed at this station in relation to bulk deposition of atmospheric mercury (Siudek et al. 2016).

In contrast, the relatively high Zn, Cu, and Pb deposition fluxes at both sites during the non-heating season (April to September) were mainly caused by favorable atmospheric conditions (e.g., low wind speed, heavy precipitation). It was previously observed for this region that the emission from coal combustion between April and September (non-heating period) is much lower than that in the other months due to reduced heating in the residential sector (Siudek et al. 2015). Therefore, other anthropogenic sources and biomass burning could have affected the atmospheric budget of TEs during the spring and summer measurements carried out within this study. Also, among the local sources that were active during the entire study period, the emission from road traffic played a significant role. The recent study by Gunawardena et al. (2013) has shown that small metallic particles (Pb, Ni, Cd, and Cu) from the exhaust emission, together with larger particles from the wear of vehicle components (Zn), had large contribution to dry and wet deposition at the sites located near heavy-traffic roads. In order to examine the influence of local traffic emission on trace element concentration in precipitation collected at the urban site, different turbulent conditions were considered. In particular, the concept of traffic-related pollution was particularly examined at low wind speed form the W-SW sectors, where two main streets were located (e.g., Dąbrowskiego Street and Saint Wawrzyńca Street). As a result, it was seen that elevated concentrations of Cu, Pb, and Cd in rainwater samples coincided with low horizontal wind speed (0–1.5 m/s) from the W-SW direction, indicating that local traffic was an important source of the aerosol containing these compounds. Conversely, during sampling days with high wind speeds (> 5 m/s), urban plumes containing trace elements of local origin could be deposited downwind of the source areas. As pointed out by Conko et al. (2004), large emission from anthropogenic sources and relatively high dispersion over megacities might give a specific scenario characterized by extremely high deposition fluxes of TEs over suburban and remote sites. It seems that similar relationships between two contrasted study domains might have taken place in the case of this study. While the anthropogenic emission from large point sources within the Poznań Agglomeration, presented in Fig. 1, directly affected the high deposition of various inorganic, metallic, and organic pollutants at the urban site, a significant influence from these sources was also found in the case of the Wielkopolski National Park in Jeziory.

In this study, a significant attention is also given on meteorological conditions during the transport of atmospheric pollutants to the sampling site. Hence, the Pb/Zn ratio was also considered as a marker of long-range transport and compared with other observations. For example, Sakata and Asakura (2009) have found that the contribution of Pb from the Asian continent to the aerosol particle concentration at the remote sites along the Japan Sea coast varied seasonally. They observed that the mean Pb/Zn ratio ranged between 0.1 and 0.3 during the warm season, increasing to 0.5 during the cold season (Sakata and Asakura 2009). In this study, we observed a remarkably similar seasonal trend for the lead-to-zinc ratio for the urban and forest sites, which was on average 0.3 and 0.2 during the cold and warm seasons, respectively (Fig. 2). Results from the present study were comparable with those obtained for the three sites along the Japan Sea coast, where a significant amount of Pb in rainwater samples was attributed to a long-range transport of this metal from distant anthropogenic sources (Sakata and Asakura 2009). Also, Moreda-Piñeiro et al. (2014) have pointed out that there was a significant influence of continental air masses on the regional concentration of trace metals in rainwater samples collected at the suburban site in Spain.

### Transport pathways of trace elements based on the backward trajectory cluster approach

**Fig. 3:**
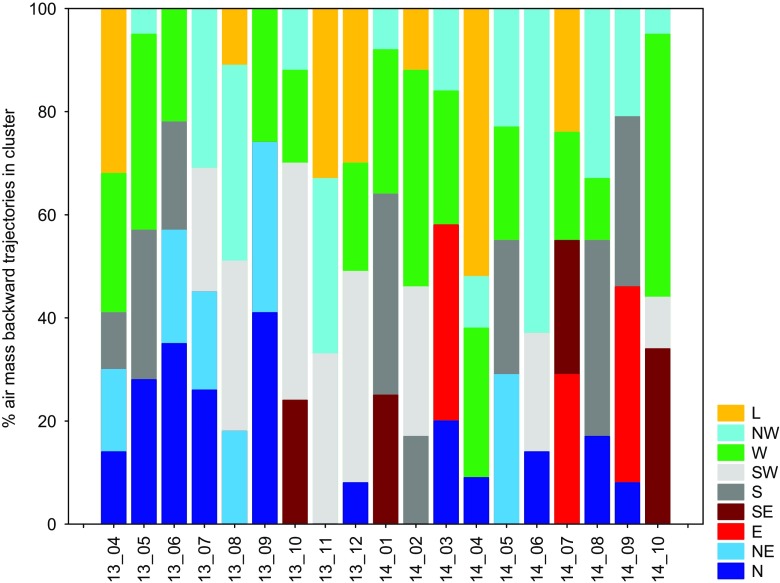
The frequency of the directions of air mass flow to the study domain (central Poland), based on backward trajectories, from April 2013 to October 2014. The cluster symbols are as follows: L local, NW northwest, W west, SW southwest, S south, SE southeast, E east, NE northeast, N north

To examine the prevalent direction of air masses over the study domain during the measurement campaign, we compared results from the cluster analysis of 2-day HYSPLIT backward trajectories. Figure 3 displays the monthly variability in the contribution of long-range transport, obtained for nine various clusters that represented the dominant advection of air masses towards the study domain. The potential source regions of TEs were quite different between seasons. In general, the N cluster was mainly associated with relatively clean air masses originating from northern European countries (Norway, Sweden, Finland), which passed over the Baltic Sea area. The NW cluster represented the air flow from northwestern European areas (UK, Ireland, Denmark, Danish Straits, Northern Germany), the North Sea, and the Atlantic Ocean, providing a mixture of maritime aerosol with gaseous and particulate compounds from various anthropogenic sources. This type of cluster was observed with high frequency during the whole study period; however, its largest contribution was found during summer months, i.e., June 2014 (63%), July 2013 (31%), August 2013 and 2014 (38 and 33%, respectively), and during fall 2013 (November, 34%). The third cluster comprised of polluted air masses from Western Europe, particularly from France, Germany, and Austria, where industrial/urban activities occurred during the whole study period. The S cluster included mainly the southern part of Europe (northern Balkans, Northern Italy, Croatia, Romania, Hungary, Czech Republic, Slovenia) plus the Upper Silesia region in Poland and some influences from the Mediterranean Sea region. According to some previous studies, the air masses associated with the S cluster are frequent during the cold period (Siudek et al. 2016). The SE cluster was associated with southeasterly transport from Ukraine and Russia and revealed moderately contribution in October 2013 and 2014, January 2014, and July 2014 (Fig. 3).

The highest contribution of the E cluster (Belarus plus Russia) was found in March 2014 (38%), September 2014 (38%), and July 2014 (29%), suggesting transport of the submicron biomass-burning aerosol and particles from different urban/industrial activities. The NE cluster was attributed mainly to the air mass advection from eastern Baltic Sea countries, i.e., Lithuania, Estonia, Latvia, and Kaliningrad districts, as well as from the areas of shipping activity in the Baltic Sea. The highest contribution from sources located within these regions was observed in September 2013 (33%) and May 2014 (29%), whereas from October 2013 to May 2014, the impact of the NE sector was potentially negligible (Fig. 3). The backward trajectory cluster analysis showed that the contribution from western advection was relatively high during October 2014, February 2014, and May 2013 and accounted for 51, 42, and 38% of the overall difference, respectively, for these months. It should be highlighted that this type of cluster was generally identified in most of the sampling seasons, indicating a significant influence of the regions in Western Europe on the aerosol composition in central Poland. As for the southwesterly transport over the study region, it was demonstrated that the anthropogenic emission from SW Europe together with regional contribution from the S-SW source areas in Poland had a significant impact on trace element distribution between October and December 2013 (on average 40%) and February 2014 (29%). In addition, the influence of SW air masses was important in summer 2013 (July 24%, August 33%) and 2014 (June 23%). As for the S cluster, the markedly high frequency (up to 33%) of southerly wind regime was found in January 2014, August 2014, and September 2014 and suggested that industrial processes within the southern sector (European sources plus regional sources) were a key factor for the atmospheric TE budget during winter and had slightly lower impact during late summer. For the local cluster (L) that reflected the regionally polluted air (SE-NE), the quite high frequency was observed during early spring (32% in April 2013 and 52% in April 2014) and winter of 2013/2014 (33% in November and 30% in December), while lower contribution was found in July 2014.

The above results support the fact that the study domain was influenced not only by local sources but also by regional emission. Moreover, the anthropogenic emission from industrial/urban activities in distant source areas of Europe together with heterogeneous processes of air pollutants in air masses can be treated as important factors affecting the seasonal variability in concentrations and deposition fluxes of trace elements in central Poland.

### Seasonal variations in TE deposition fluxes vs. air mass transport sectors

Table 3 shows the results of cluster-based approach in relation to the bulk deposition fluxes of seven trace elements at the urban site, between April 2013 and October 2014. In general, the seasonal differences in deposition fluxes of trace elements were evident in each cluster, indicating a significant impact of different source areas. The bulk deposition fluxes of trace elements associated with southern sectors (SW-SE) were much higher than those attributed to the northern ones (NE-NW), during the entire sampling period. The mean deposition fluxes of Cu, Ni, and Pb related to the N cluster ranged between 2.07 and 60.4 μg m^−2^, between 0.54 and 111.3 μg m^−2^, and between 1.64 and 30.0 μg m^−2^, respectively. In contrast to the aforementioned N cluster, higher deposition fluxes of Zn, As, Cd, and Cr were observed for the S cluster (23.7–142.0, 0.44–15.1, 0.08–0.91, and 0.96–19.0 μg m^−2^ event^−1^, Table 3). Furthermore, previous studies by Siudek et al. (2015, 2016) have shown that during the warm study period in Poznań, the particulate-phase pollutants originated mainly from anthropogenic sources (urban/industrial processes) and traffic, while during fall and winter months, such source as domestic heating from the local residential sector had an additional influence. As shown in Table 3, in spring 2013, the mean deposition fluxes for almost all variables, except for Cd, reached the highest values during the southerly advection pathways, while the lowest values were mostly attributed to the N cluster. For example, in spring 2013, mean Zn deposition fluxes associated with southerly transport pattern were on average about 5–126% higher than those observed in westerly and northeasterly clusters. Slightly different deposition trends were observed during spring 2014, when (1) peak values of the deposition fluxes for Cr, Pb, Cu, and Zn were registered in the western cluster; (2) higher As and Cd values were attributed to the eastern cluster; and (3) higher Ni fluxes were found in the southern cluster. In this study, mean Cd deposition fluxes were highly variable (0.04–0.75 μg m^−2^ event^−1^), with significantly higher values in the NE cluster observed during spring and fall 2013, indicating relatively high precipitation depth and high emission of this metal from industrial activities in multiple urban areas of northeastern Poland (chemical plants) and Europe (e.g., Pb smelter and cement plant in Estonia; Ni/Cu and Cd smelters in Harjavalta region, southwestern Finland; Zn smelter and paper plants in Kokkola region, Finland; Ni smelters in Russia). As noted above, the observed seasonal discrepancies can be attributed to the differences in concentration levels and precipitation depth. Similar relationships between deposition fluxes and precipitation depth have been documented by other authors (Sakata and Asakura 2009; Kara et al. 2014; Pan and Wang 2015).

**Table 3:** Mean bulk deposition fluxes of trace elements (μg m^−2^ event^−1^) assigned to one of the nine clusters extracted from the air mass backward trajectory analysis, with total precipitation depth (mm) for the sampling seasons in Poznań.

Season (month of sampling)	Variables	N	NE	E	SE	S	SW	W	NW	L
Spring 13 (13_04, 13_05)	As	0.13	0.64			**3.81**		1.16	0.53	1.57
	Cd	0.04	**0.75**			0.17		0.07	0.02	0.04
	Ni	0.54	0.73			**8.05**		4.03	3.99	3.56
	Cr	0.27	0.81			**19.0**		0.91	1.25	0.59
	Pb	1.64	0.93			**22.7**		5.60	2.22	5.56
	Cu	2.07	1.02			**13.9**		7.40	5.23	3.53
	Zn	22.0	1.13			**142.0**		32.1	20.0	19.9
Precipitation (mm)	*88.90*									
Summer 13 (13_06, 13_07, 13_08)	As	0.85	1.03			0.44	6.44	0.34	1.32	**3.69**
	Cd	0.09	0.3			0.08	**0.52**	0.13	0.08	0.15
	Ni	2.29	12.1			2.25	11.7	6.00	7.74	**14.8**
	Cr	0.44	3.64			1.51	**6.26**	2.28	0.68	0.38
	Pb	3.42	43.1			8.12	**55.2**	10.9	3.00	13.16
	Cu	4.81	23.6			7.91	**36.0**	13.5	4.84	4.62
	Zn	26.2	127.6			23.7	**244.4**	35.6	27.3	116.6
Precipitation (mm)	*180.3*									
Fall 13 (13_09, 13_10, 13_11)	As	4.03	**29.5**		2.96		7.48	2.51	4.62	10.4
	Cd	0.06	**0.47**		0.45		0.48	0.13	0.15	0.21
	Ni	7.47	**23.9**		7.06		22.3	9.62	8.00	11.1
	Cr	1.13	2.72		**2.93**		1.46	0.85	1.09	1.45
	Pb	16.1	18.8		**34.9**		22.8	4.85	8.68	14.9
	Cu	6.88	25.7		**63.0**		32.6	12.7	19.9	26.8
	Zn	41.7	6.00		**127.9**		76.7	28.4	41.7	74.4
Precipitation (mm)	*128.2*									
Winter 13/14 (13_12, 14_01, 14_02)	As	3.85			16.0	2.61	4.85		**13.6**	7.14
	Cd	0.41			**0.72**	0.17	0.08		0.44	0.21
	Ni	3.73			**33.8**	14.8	0.72		7.11	6.71
	Cr	1.47			**2.30**	0.96	0.72		1.69	1.24
	Pb	19.9			**94.8**	19.4	6.78		28.5	21.4
	Cu	18.9			17.6	21.6	10.6		**32.7**	22.1
	Zn	50.8			**128.0**	42.3	22.7		78.1	44.3
Precipitation (mm)	*84.6*									
Spring 14 (14_03, 14_04, 14_05)	As	2.19	3.30	**15.0**		1.09		8.58	6.13	
	Cd	0.26	0.22	**0.60**		0.17		0.56	0.33	
	Ni	6.88	7.86	10.8		**23.6**		11.6	12.5	
	Cr	1.34	1.17	1.40		1.15		**6.00**	2.25	
	Pb	7.93	3.63	18.6		6.09		**48.1**	18.3	
	Cu	22.6	3.61	36.2		22.3		**110.5**	58.3	
	Zn	78.2	7.19	111.4		66.4		**151.4**	84.6	
Precipitation (mm)	*198.6*									
Summer 14 (14_06, 14_07, 14_08)	As	3.82		0.98		**15.1**	10.01	6.09	2.26	5.33
	Cd	0.12		**0.94**		0.91	0.37	0.06	0.09	0.27
	Ni	4.31		32.3		57.5	**71.9**	18.2	3.32	14.8
	Cr	0.78		**3.24**		2.20	1.88	1.14	1.01	1.10
	Pb	3.50		**36.3**		17.8	6.32	7.76	9.19	8.34
	Cu	6.11		**165.8**		50.7	30.0	42.2	30.7	8.99
	Zn	21.9		41.5		**100.8**	83.4	60.8	44.4	38.8
Precipitation (mm)	*176.4*									
Fall 14 (14_09, 14_10)	As	4.88		7.11	0.24	1.73	**14.7**	1.18	1.05	
	Cd	0.19		0.26	0.01	0.12	**0.40**	0.08	0.03	
	Ni	**111.3**		56.8	3.48	8.96	21.1	61.7	8.02	
	Cr	2.40		**6.14**	0.21	2.31	2.40	1.00	0.38	
	Pb	30.0		**61.1**	0.60	13.5	27.6	4.36	1.69	
	Cu	60.4		**208.4**	18.1	41.6	49.4	11.0	32.6	
	Zn	91.7		**137.0**	27.6	73.6	127.5	28.2	46.1	
Precipitation (mm)	*86.3*									

The bold data represent the highest values of deposition fluxes

The precipitation depth measured in each season is presented in italics

Among the clusters selected in this study, the highest mean Cu deposition flux was observed for the E sector during summer 2014 (165.8 μg m^−2^ event^−1^) and fall 2014 (208.4 μg m^−2^ event^−1^). In contrast, an extremely high Ni deposition flux of 100.8 μg m^−2^ event^−1^ occurred in fall 2014 and was associated with industrial activities related to northern areas (N cluster). Additionally, the mean Pb deposition flux for the SE cluster during winter 2013/2014 (94.8 μg m^−2^ event^−1^) was significantly higher than that for the other clusters and seasons, suggesting large contribution from local/regional anthropogenic sources and long-range transport. The deposition fluxes of As and Ni associated with the L cluster were higher in comparison to the remaining clusters only in spring 2013, indicating a significant impact of the regional sources directly related to industrial coal combustion on trace element concentrations and deposition fluxes.

## Conclusions

The long-term measurement campaign focused on atmospheric transport and deposition of trace elements was performed in central Poland between April 2013 and October 2014. This study allowed to draw the following conclusions: (1) mean bulk deposition fluxes of trace metals were higher at the urban site compared with the forest site, and (2) peak values of TE depositions at two sampling sites were observed in different seasons. Moreover, the results from this study allowed to identify constant local and regional influences on trace elements in precipitation over central Poland, while the trajectory cluster analysis provided an important insight into the dynamics of atmospheric processes in the urban environment. The range of TE deposition fluxes in Poznań was consistent with the values registered in other polluted sites and indicated a significant impact of urban sources on the rainwater chemistry. In this study, the mean deposition values of Zn, Cu, Ni, Pb, As, Cd, and Cr associated with the N cluster were on average in the following ranges: 21.9–91.7, 2.07–60.4, 0.54–111.3, 1.64–30.0, 0.13–4.88, 0.04–0.41, and 0.27–2.40 μg m^−2^ event^−1^, whereas fluxes of these metals associated with the S cluster ranged as follows: 23.7–142.0, 7.91–50.7, 2.25–57.5, 6.09–22.7, 0.44–15.1, 0.08–0.91, and 0.96–19.0 μg m^−2^ event^−1^. It was showed that the long-range transport may be considered as an additional source of trace elements in rainwater during the whole study period. Besides the important information for the networks monitoring the air quality, these results can become useful in similar observations and modeling studies aimed at better quantification of the TE budget in the polluted urban atmosphere and to make the comprehensive assessment of soil/water system contamination and risk for human health.
